# A new polystomatid (Monogenea, Polystomatidae) from the mouth of the North American freshwater turtle *Pseudemys nelsoni*

**DOI:** 10.3897/zookeys.539.6108

**Published:** 2015-11-23

**Authors:** Louis H. Du Preez, Michelle Van Rooyen

**Affiliations:** 1 Unit for Environmental Sciences and Management, North-West University, Potchefstroom, 2520, South Africa

**Keywords:** Monogenea, Polystomatidae, *Polystomoides*, freshwater turtle, Florida, USA

## Abstract

Based on material collected from *Pseudemys nelsoni* (Reptilia: Chelonia: Emydidae) during a parasite survey of the herpetofauna around Gainesville, Florida, USA, *Polystomoides nelsoni* **sp. n.** is described as a new polystome species. This parasite was found in the oral and pharyngeal region of the host. In a sample of nine *Pseudemys nelsoni*, three specimens were found to release polystome eggs. One turtle was euthanized and dissected and found to be infected in the oral region with 19 specimens belonging to an as-yet-unknown *Polystomoides*. This is only the fifth *Polystomoides* recorded from the Nearctic realm. This species is distinguished from known species by a combination of characteristics including marginal hooklet morphology, body length and haptor dimensions.

## Introduction

Although monogeneans are predominantly single host fish parasites, polystomatid flatworms (Monogenea, Polystomatidae) radiated onto the tetrapods and are known from a diverse range of hosts, including the Australian lungfish, amphibians, freshwater turtles and the hippopotamus (Raharivololoniaina et al. 2011). Of the 24 currently known polystome genera (Du Preez et al. 2014) three exclusively parasitize turtles, namely *Polystomoides* Ward, 1917, *Polystomoidella* Price, 1939 and *Neopolystoma* Price, 1939 (see Du Preez and Moeng 2004, Du Preez et al. 2007, Du Preez et al. 2008, Morrison and Du Preez 2012, Price 1939, Wright 1879, Yamaguti 1963).

Genera of the subfamily Polystomoidinae, including the three polystome genera known from turtles and the genus *Nanopolystoma*, known from caecilians (Du Preez et al. 2008), all have non-confluent gut caeca lacking diverticula and possess skeletal elements inside the haptoral suckers. Chelonian polystomes are further characterised by non-diverticulated gut caeca of equal length and subsequent, absence of prehaptoral or haptoral anastomoses and a compact medial testis. *Polystomoides* is found in the oral cavity, nasal cavity, cloaca and urinary bladder of the host and has two pairs of hamuli, with the outer pair being larger than the inner pair. *Polystomoidella* parasitizes the urinary bladder of turtles and has a single pair of hamuli. *Neopolystoma* is found in the oral cavity, nasal cavity, ocular cavity, cloaca and urinary bladder and has no hamuli.

At present 54 turtle polystome species are known from 55 host species. Although chelonian polystomes have a broad geographical distribution, only seven *Neopolystoma*, two *Polystomoidella* and four *Polystomoides* species are known from the Nearctic realm. The *Polystomoides* species currently known from this region include *Polystomoides coronatum* (Leydi, 1888) Ozaki, 1935 from *Trachemys dorbigni*; *Polystomoides multifalx* Stunkard, 1924 from *Pseudemys concinna* (LeConte, 1830); *Polystomoides oris* Paul, 1938 and *Polystomoides pauli* Timmers & Lewis, 1979, both from *Chrysemys picta*.

During a survey of freshwater turtles around Gainesville, Florida, USA, *Pseudemys nelsoni* (Reptilia: Chelonia: Emydidae) was found to be infected with an as-yet-unknown *Polystomoides*. This paper provides the formal description of this previously unknown parasite.

## Material and methods

During April-June 2004 baited crayfish traps were set to capture terrapins in ponds in and around Gainesville, Florida, USA. Captured turtles were individually placed in 20 L plastic buckets with dechlorinated tap water to a depth of about 50 mm. After a period of 24 hours turtles were removed and the water screened for the presence of polystome eggs. The water from the containers in which turtles were housed was poured through two plankton sieves with respective mesh sizes of 500 µm and 100 µm. The first sieve removed the coarse debris in the water while the second retained finer debris and any polystome eggs that might be present. The contents of both sieves were then washed into separate glass Petri dishes and examined under a dissecting microscope. The Petri dish with contents from the course sieve was scanned for adult parasites that may have dislodged, and the Petri dish with contents from the fine sieve was scanned for polystome eggs.

Recovered eggs were removed and incubated at room temperature in Petri dishes containing clean water. Freshly hatched oncomiracidia were collected and mounted semi-permanently using ammonium picrate as mounting medium to clear the parasites and reveal the marginal hooklets. Turtles that were found not to be infected with polystome eggs were screened a second and third period of 24 hours. A single infected turtle was euthanized by injecting 0.5 mL of sodium pentobarbitone diluted with water (0.5 mL pentobarbitone and 4.5 mL water) straight into the heart. After 15 minutes the specimen was dissected. The cloaca, urinary bladder and accessory bladders as well as the oral cavities, nasal cavities, pharyngeal cavities, eye surface and cavity under the nictitating membrane were examined for polystomes, with the aid of a stereo microscope. The remainder of the turtles were released where collected.

Polystome whole mounts were prepared as follows: individual mature polystome species collected from the host species were fixed under cover-slip pressure in 10% neutral buffered formalin (NBF). Representative sub-adult specimens were fixed in 70% molecular grade ethanol for future molecular studies.

Parasites earmarked for permanent mounts were hydrated using 30% EtOH, stained overnight in a weak aceto-carmine staining solution, gradually dehydrated to absolute EtOH, cleared in a 1:1 ratio mix of absolute ethanol-xylene and then pure xylene, and mounted in Canada balsam. Preparations were studied using a Nikon E800 compound microscope fitted with a Nikon DXM1200 digital microscope camera connected to a PC. Measurements were taken using Eclipse network software (Nikon). Marginal hooklet measurements were obtained from the oncomiracidia that hatched from incubated eggs, following the protocol developed by Du Preez and Maritz (2006).

## Results

### Turtles screened and polystomes retrieved

Nine Florida red-bellied turtles (*Pseudemys nelsoni*) were collected and screened. Specimens were collected from Lake Griffin, Lake Lochloosa, Lake Orange, and ponds at the U.S. Geological Survey (USGS) research facility in Gainesville.

### Levels of infection

Of the nine turtles examined three were found to be infected (prevalence 33%). Only one turtle was dissected and found to have 19 polystomes in the oral region. These specimens were identified as belonging to *Polystomoides*; however, they did not conform to any of the 38 known *Polystomoides* species.

### Molecular studies

Material collected was also studied at the molecular level. Based on 18S and 28S rDNA sequences, the newly discovered polystome differs from all other known turtle polystomes for which molecular data are available and occupies a distinct position basal to other Nearctic chelonian polystomes (see Figures 2a and 2b in Verneau et al. 2011).

## Systematics

### Class: Monogenea Carus, 1863 Order: Polystomatidea Lebedev, 1988 Family: Polystomatidae Gamble, 1896

#### Polystomoides nelsoni sp. n.

Taxon classificationAnimaliaPolystomatideaPolystomatidae

http://zoobank.org/757AA55C-4C80-4075-9B57-A297833F70DA

Figs 1 , 2

##### Specimens studied.

Morphological description based on ten sexually mature worms. Holotype (NMB 380) nine paratypes (NMB 381–389) deposited in the Parasitic Worm Collection, National Museum, Aliwal Street, Bloemfontein, South Africa.

##### Type host.

*Pseudemys nelsoni* (Carr, 1938) sexually mature male.

##### Type locality.

United States Geological Survey USGS-BRD facility, 7920 N.W. 71st St., Gainesville, Florida, USA (29°43'31"N, 82°25'04"W).

##### Etymology.

The species is named after the host.

##### Site.

Mouth.

##### Description.

Based on ten egg-producing adults. The average measurement is given, followed by the range given in parentheses. Measurements are given in micrometres (µm). Larval (oncomiracidia) measurements are given for the marginal hooklets.

**Figure 1.**
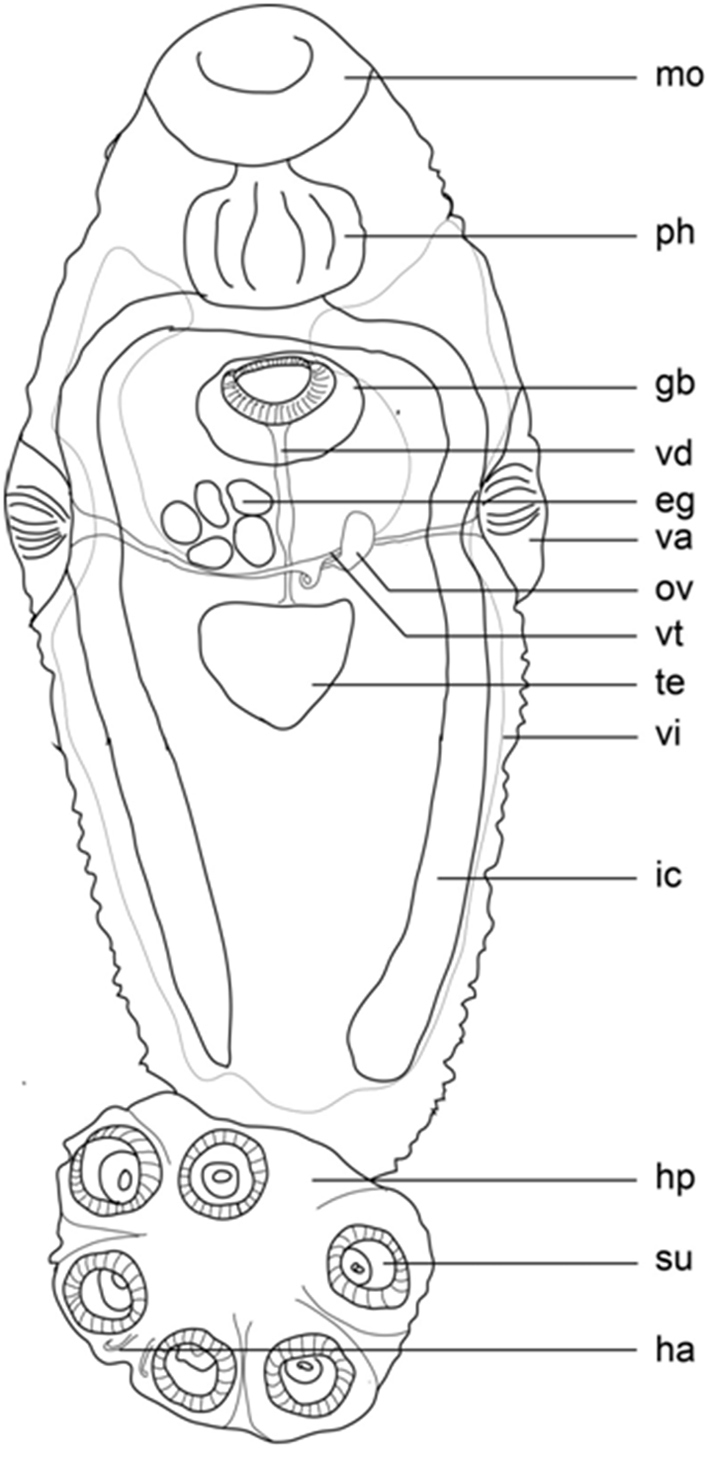
*Polystomoides nelsoni* sp. n. Ventral view of holotype; the dotted line indicates the outline of the vitellarium. Abbreviations: eg, egg; gb, genital bulb; ha, hamulus; hp, haptor; ic, intestinal caecum; mo, mouth; ov, ovary; ph, pharynx; su, sucker; te, testis; va, vagina; vd, vas deferens; vi, vitellarium; vt, vitelline duct. Scale bar: 1 mm.

**Figure 2.**
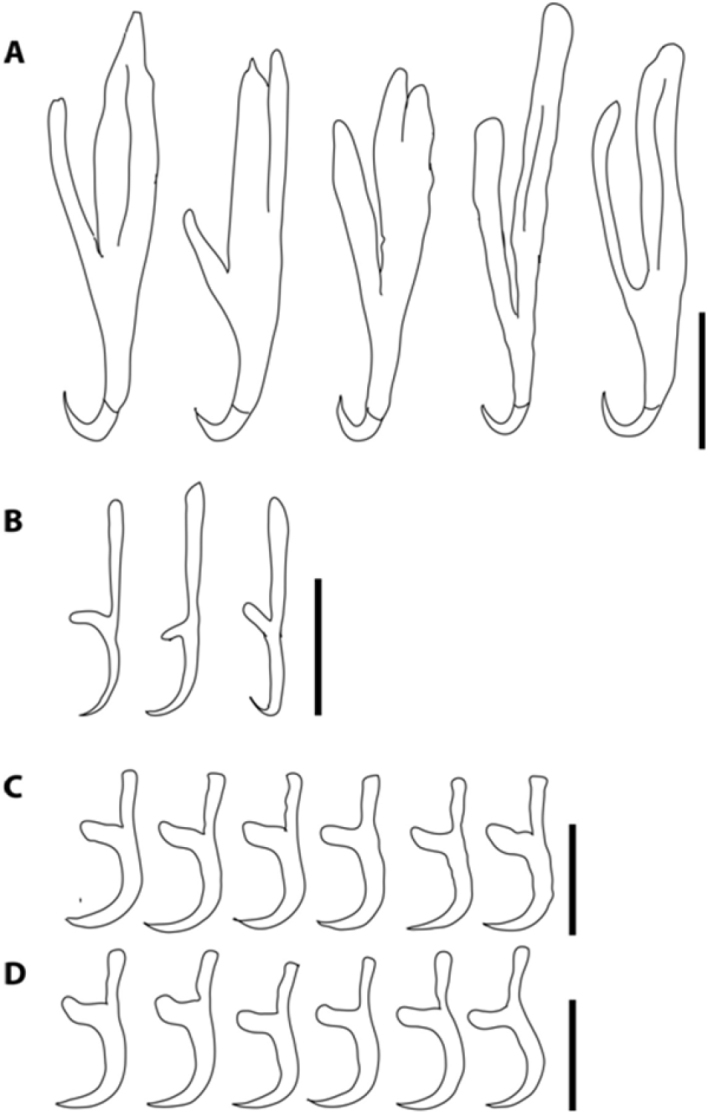
*Polystomoides nelsoni* sp. n. **A** large hamuli from the holotype and paratypes **B** small hamuli from holotype and paratype **C** marginal hooklets 1 **D** marginal hooklets 2–8. Scale bars: 50 µm (**A, B**); 20 µm (**C, D**).

*Adult*: General characteristics given of mature, egg-producing parasite (Figure 1). Body elongated and ellipsoid, total length 5.707 (3.052–7.378), greatest width 2.278 (1.276–2.751), width at vagina 2.270 (1.276–2.739), haptor length 1.310 (912–1.616), haptor width 1.931 (1.232–2.182); haptor length to body length ratio 0.23; six haptoral suckers, mean diameter 564 (148–781), haptors internally supported by an elaborate skeletal structure. Two pairs of hamuli: inner pair 69 (48–95) long with a hamulus hook length of 21 (17–26); outer pair 138 (104–173) long with a hamulus hook length of 22 (19–26). Mouth sub-terminal. False oral sucker 788 (398–1 036) wide; pharynx length 539 (345–917), width 658 (391–881). Intestine bifurcates with no diverticula and no anastomoses present; caeca extend to the end of the body proper and do not join posteriorly nor do they extend into the haptor. Testis compact, mid-ventral, medial, and posterior to ovary (Figure 1); 401 (108–687) long and 564 (148–781) wide. Genital atrium median, ventral, posterior to intestinal bifurcation: 586 (302–816) in length with 123 (108–132) spines, 101 (93–106) long. Ovary, dextral, anterior, 38% of body length; ovary length 251 (102–330), and width 86 (27–124). Short tubular uterus anterior to ovary, containing up to eight eggs; length 227 (182–274), and width 144 (118–194). No intra-uterine development, operculated egg. Vitellarium extends throughout most of the body proper posterior to the pharynx except the central area around the gonads (Figure 1). **Oncomiracidia.** Marginal hooklets were observed and measured on slides prepared from incubated oncomiracidia (Figure 2). Marginal hooklet I found to be 28 (25–30) and hooklets II – VIII 27 (25–29).

##### Remarks.

*Polystomoides nelsoni* sp. n. differs from other *Polystomoides* species by a combination of characters. With a body length of 5.707 (3.052–7.378), *Polystomoides nelsoni* sp. n. is longer than *Polystomoides megaovum* (2.910), *Polystomoides asiaticus* (4.600), *Polystomoides siebenrockiella* (3.580) and *Polystomoides uruguayensis* (2.560–2.650). However, *Polystomoides nelsoni* sp. n. has a shorter body length when compared to *Polystomoides australiensis* (6.193), *Polystomoides fuquesi* (7.480–7.550), *Polystomoides godavarii* (4.200–8.030) and *Polystomoides ludhianae* (6.640–10.060). In terms of the length and width of the haptor, *Polystomoides nelsoni* sp. n. (1.310 × 1.931) differs from *Polystomoides megaovum* (620 × 880), *Polystomoides asiaticus* (1.100 × 1.700), *Polystomoides siebenrockiella* (780 × 1.060), *Polystomoides australiensis* (1.353 × 2.190) and *Polystomoides godavarii* (1.120–1.620 × 1.250–1.710).

## Discussion

All polystome species are host-specific, with chelonian polystomes being strictly site-specific. As a result of this strict site specificity a single host could be infected by more than one polystome species. Chelonian polystomes have been fairly well studied in the USA, with 11 polystomes known from various freshwater turtle hosts (Du Preez and Lim 2000, Morrison and Du Preez 2012).

The two *Polystomoidella* species known from North America are *Polystomoidella oblongum* Wright, 1879 and *Polystomoidella whartoni* Wright, 1879. The seven *Neopolystoma* species known from the USA are: *Neopolystoma elizabethae* Platt, 2000; *Neopolystoma fentoni* Platt, 2000; *Neopolystoma grossi* Morrison & Du Preez, 2012; *Neopolystoma moleri* Morrison & Du Preez, 2012; *Neopolystoma orbiculare* Stunkard, 1916; *Neopolystoma rugosa* MacCallum, 1918; and *Neopolystoma terrapenis* Harwood, 1932. The four *Polystomoides* species known from the USA are: *Polystomoides coronatum* Leidy, 1888; *Polystomoides multifalx* Stunkard, 1924; *Polystomoides oris* Paul, 1938; and *Polystomoides pauli* Timmers & Lewis, 1979 (Morrison and Du Preez 2012).

The main feature distinguishing *Polystomoides* from other turtle polystomes is the presence of two unequal pairs of hamuli. The other genera that parasitize turtles either have a single pair of hamuli as in *Polystomoidella* or the hamuli are lacking altogether as in *Neopolystoma. Polystomoides* and *Neopolystoma* species can also occasionally be distinguished from *Polystomoidella* in terms of the additional sites (the cavity of the eye and nose, pharynx, cloaca, and mouth) that these species parasitize, as *Polystomoidella* parasites are found to infect only the urinary bladder of their host species.

*Polystomoides nelsoni* sp. n. can be distinguished from the other *Polystomoides* species by the number of genital spines. *Polystomoides nelsoni* sp. n. has 123 (108–132) genital spines compared to *Polystomoides fuquesi* with 2, *Polystomoides brasiliensis* with 8–9, *Polystomoides bourgati* with 26–29, *Polystomoides asiaticus* with 34–40, *Polystomoides ludhianae* with 54–64, *Polystomoides godavarii* with 64–66, and *Polystomoides australiensis* with 74–95. However, *Polystomoides multifalx* (120–124) and *Polystomoides stunkardi* (92–109) are two species that also have a large number of genital spines. Compared to *Neopolystoma* species, *Polystomoides nelsoni* sp. n. also has a larger number of genital spines. *Neopolystoma chelodinae* has 14 (12–16), *Neopolystoma elizabethae* 8 and *Neopolystoma euzeti* 34 (33–36), while *Polystomoides oblongum* and *Polystomoides whartoni* both have 16 genital spines.

The total length of the genital spines of *Polystomoides nelsoni* sp. n. 101 (93–106) is longer compared to those of other *Polystomoides* species, such as *Polystomoides siebenrockiella* 58 (54–60), *Polystomoides rohdei* 34–52, *Polystomoides platynota*, 60–70, *Polystomoides nabedei* 42–46, *Polystomoides microrchis* 75–88 and *Polystomoides chabaudi* 27 (22–31). The genital spines for *Polystomoides nelsoni* sp. n. are in the same size range as those of *Polystomoides australiensis* 93 (78–105). *Polystomoides nelsoni* sp. n. also has larger genital spines compared to those of *Neopolystoma* species, such as *Neopolystoma chelodinae* 23.6 (20.8–27.2), *Neopolystoma euzeti* 57 and *Neopolystoma elizabethae* 10, as well as compared to those of *Polystomoidella* species, such as *Polystomoides oblongum* 18–22 and *Polystomoides whartoni* 15–18.

Unlike most other polystomes, these parasitizing chelonians have a broad geographical distribution. Both *Neopolystoma* and *Polystomoides* have been reported from the realms around the globe known to be inhabited by freshwater turtles. On the other hand, *Polystomoidella* is mainly known from the Nearctic realm where it is represented by five species. However, Richardson and Brooks (1987) described *Polystomoidella mayesi* from the urinary bladder of a Malaysian box turtle, *Cuora amboinensis*. The presence of *Polystomoidella* in the Oriental realm raises questions of possible misidentifications or a possible parasite transfer. According to Du Preez and Lim (2000) the possibility of transfer from an introduced American turtle can only be confirmed or refuted if and when *Polystomoides mayesi* is found in this chelonian species.

Part of the evolutionary success of chelonian polystomes is the fact that they are site-specific and occupy various sites, including the oral and nasal cavities, eye cavity and the cloaca and urinary bladder. Littlewood et al. (1997) stated that congeneric species infecting the same site in different hosts are more closely related than congeneric species infecting different sites in the same host individuals. The high degree of site specificity allows for speciation and could explain the polystome diversity found in freshwater turtles. With the huge diversity of freshwater turtles globally it is likely that a vast number of chelonian polystomes remain to be discovered.

## Supplementary Material

XML Treatment for Polystomoides nelsoni
